# Supplementary material for: Changing trends of corporate social responsibility reporting in the world-leading airlines
Source: PLoS One. 2020 Jun 8;15(6):e0234258. doi: 10.1371/journal.pone.0234258 (PMC7279591; doi:10.1371/journal.pone.0234258)
Supplement: S1 Appendix — (DOCX) [file pone.0234258.s004.docx]

**Appendix 1. The selected airlines, reporting year and word count of the CSR reports**

| **Airlines** | **County/ Region** | **Alliance** | **Year** | **Word count of report** |
| --- | --- | --- | --- | --- |
| Aeroflot | Russia | Skyteam | 2013 | 84,635 |
|  |  |  | 2014 | 87,894 |
|  |  |  | 2015 | 102,711 |
|  |  |  | 2016 | 102,711 |
|  |  |  | 2017 | 110,135 |
| Air Canada | Canada | Star Alliance | 2013 | 18,667 |
|  |  |  | 2014 | 19,995 |
|  |  |  | 2015 | 23,814 |
|  |  |  | 2016 | 23,988 |
|  |  |  | 2017 | 994 |
| Air China | China | Star Alliance | 2013 | 20,956 |
|  |  |  | 2014 | 24,967 |
|  |  |  | 2015 | 66,772 |
|  |  |  | 2016 | 26,347 |
|  |  |  | 2017 | 15,982 |
| Air France-KLM | France, Netherland | Skyteam | 2013 | 33,720 |
|  |  |  | 2014 | 35,006 |
|  |  |  | 2015 | 152,752 |
|  |  |  | 2016 | 153,752 |
|  |  |  | 2017 | 27,434 |
| Alitalia | Italy | Skyteam | 2013 | 126,869 |
|  |  |  | 2014 | 133,585 |
|  |  |  | 2015 | 131,557 |
|  |  |  | 2016 | 137,228 |
|  |  |  | 2017 | 139,025 |
| American Airline | America | Oneworld | 2013 | 860 |
|  |  |  | 2014 | 8,624 |
|  |  |  | 2015 | 15,722 |
|  |  |  | 2016 | 10,808 |
|  |  |  | 2017 | 15,875 |
| All Nippon Airways (ANA) | Japan | Star Alliance | 2013 | 72,280 |
|  |  |  | 2014 | 50,500 |
|  |  |  | 2015 | 55,632 |
|  |  |  | 2016 | 66,067 |
|  |  |  | 2017 | 67,662 |
| Cathay Pacific | Hong Kong | Oneworld | 2013 | 19,818 |
|  |  |  | 2014 | 19,872 |
|  |  |  | 2015 | 20,149 |
|  |  |  | 2016 | 34,264 |
|  |  |  | 2017 | 22,688 |
| China Airlines | Taiwan | Skyteam | 2013 | 39,147 |
|  |  |  | 2014 | 55,277 |
|  |  |  | 2015 | 63,162 |
|  |  |  | 2016 | 43,262 |
|  |  |  | 2017 | 50,303 |
| Croatia Airlines | Croatia | Star Alliance | 2013 | 34,165 |
|  |  |  | 2014 | 33,697 |
|  |  |  | 2015 | 33,161 |
|  |  |  | 2016 | 35,429 |
|  |  |  | 2017 | 16,661 |
| Delta Air Lines | America | Skyteam | 2013 | 24,453 |
|  |  |  | 2014 | 26,940 |
|  |  |  | 2015 | 41,902 |
|  |  |  | 2016 | 31,307 |
|  |  |  | 2017 | 29,694 |
| Ethiopia Airlines | Ethiopia | Star Alliance | 2013 | 11,632 |
|  |  |  | 2014 | 14,322 |
|  |  |  | 2015 | 11,254 |
|  |  |  | 2016 | 15,480 |
|  |  |  | 2017 | 10,233 |
| Finnair | Finland | Oneworld | 2013 | 93,965 |
|  |  |  | 2014 | 95,073 |
|  |  |  | 2015 | 16,162 |
|  |  |  | 2016 | 91,181 |
|  |  |  | 2017 | 94,580 |
| Garuda Indonesia | Indonesia | Skyteam | 2013 | 109,890 |
|  |  |  | 2014 | 35,460 |
|  |  |  | 2015 | 38,997 |
|  |  |  | 2016 | 52,096 |
|  |  |  | 2017 | 49,973 |
| Japan Airlines | Japan | Oneworld | 2013 | 41,956 |
|  |  |  | 2014 | 45,978 |
|  |  |  | 2015 | 42,606 |
|  |  |  | 2016 | 63,988 |
|  |  |  | 2017 | 58,442 |
| Korean Air | Korea | Skyteam | 2013 | 34,048 |
|  |  |  | 2014 | 23,777 |
|  |  |  | 2015 | 36,340 |
|  |  |  | 2016 | 27,853 |
|  |  |  | 2017 | 27,338 |
| Latam Airlines | Chile | Oneworld | 2013 | 28,592 |
|  |  |  | 2014 | 18,491 |
|  |  |  | 2015 | 25,340 |
|  |  |  | 2016 | 29,875 |
|  |  |  | 2017 | 31,082 |
| Lufthansa | German | Star Alliance | 2013 | 44,919 |
|  |  |  | 2014 | 51,376 |
|  |  |  | 2015 | 44,407 |
|  |  |  | 2016 | 46,198 |
|  |  |  | 2017 | 47,916 |
| Scandinavian Airlines (SAS) | Sweden | Star Alliance | 2013 | 28,564 |
|  |  |  | 2014 | 27,035 |
|  |  |  | 2015 | 16,401 |
|  |  |  | 2016 | 16,344 |
|  |  |  | 2017 | 17,447 |
| Singapore Airlines | Singapore | Star Alliance | 2013 | 17,617 |
|  |  |  | 2014 | 20,653 |
|  |  |  | 2015 | 23,678 |
|  |  |  | 2016 | 24,314 |
|  |  |  | 2017 | 27,202 |
| Turkish Airlines | Turkey | Star Alliance | 2013 | 3,001 |
|  |  |  | 2014 | 28,537 |
|  |  |  | 2015 | 26,373 |
|  |  |  | 2016 | 7,278 |
|  |  |  | 2017 | 25,880 |
|  |  |  | Total count of words | 4,662,021 |
