# Supplementary material for: Changing trends of corporate social responsibility reporting in the world-leading airlines
Source: PLoS One. 2020 Jun 8;15(6):e0234258. doi: 10.1371/journal.pone.0234258 (PMC7279591; doi:10.1371/journal.pone.0234258)
Supplement: S2 Appendix — (DOCX) [file pone.0234258.s005.docx]

**Appendix 2. The selected keywords in the sub-topics of economic issues**

| **201** | **202** | **203** | **204** | **205** | **206** |
| --- | --- | --- | --- | --- | --- |
| financial | wage | impacts | supplier | corruption | competitive |
| benefits | market | indirect | practices | incidents | behavior |
| government | level | economy | local | risk | monopoly |
| costs | presence | investments | procurement | governance | trust |
| accounts | time | infrastructure | economic | action | collusion |
| opportunities | entry | supported | chain | bribery | effects |
| payments | full |  |  |  | geographic |
|  |  |  |  |  | price |
