# Supplementary material for: Changing trends of corporate social responsibility reporting in the world-leading airlines
Source: PLoS One. 2020 Jun 8;15(6):e0234258. doi: 10.1371/journal.pone.0234258 (PMC7279591; doi:10.1371/journal.pone.0234258)
Supplement: S3 Appendix — (DOCX) [file pone.0234258.s006.docx]

**Appendix 3. The selected keywords in the sub-topics of environmental issues**

| **301** | **302** | **303** | **304** | **305** | **306** | **307** | **308** |
| --- | --- | --- | --- | --- | --- | --- | --- |
| materials | energy | water | biodiversity | emissions | waste | compliance | environmental |
| renewable | sources | discharge | protected | GHG | disposal | laws | assessments |
| packaging | fuel | stress | species | protocol | hazardous | references | negative |
| recycled | reduction | quality | habitats | CO2 | effluents | related | expectations |
| weight | activities | withdrawal | list | GWP | transported | applicable | potential |
| volume | consumed | treatment | conservation | rates | spills | disclosures | directly |
| manufacturing | intensity | consumption | natural | type | destination | authorities | mitigate |
| parts | outside | clause | affected | air | methods | approach |  |
|  | gas |  |  | scope |  |  |  |
