# Supplementary material for: Changing trends of corporate social responsibility reporting in the world-leading airlines
Source: PLoS One. 2020 Jun 8;15(6):e0234258. doi: 10.1371/journal.pone.0234258 (PMC7279591; doi:10.1371/journal.pone.0234258)
Supplement: S4 Appendix — (DOCX) [file pone.0234258.s007.docx]

**Appendix 4. The selected keywords in the sub-topics of social issues**

| **401** | **402** | **403** | **404** | **405** | **406** | **407** | **408** | **409** | **410** | **411** | **412** | **413** | **414** | **415** | **416** | **417** | **418** | **419** |  |
| --- | --- | --- | --- | --- | --- | --- | --- | --- | --- | --- | --- | --- | --- | --- | --- | --- | --- | --- | --- |
| employee | | international | work | training | diversity | discrimination | collective | child | forced | security | peoples | rights | community | social | political | customer | marketing | privacy | socioeconomic |
| employed | | operations | health | education | equal | forms | bargaining | labour | product | personnel | indigenous | human | engagement | criteria | public | safety | labeling | data | regulations |
| working | | relations | workers | skills | nations | person | association | age | compulsory | conduct | informed | declaration | stakeholder | supply | policy | period | communication | information | area |
| leave | | employment | occupational | programs | remuneration | effective | freedom | minimum | convention | third | sustainability | agreements | groups | provide | contributions | concerning | background | protection | environment |
| legally | | changes | injuries | review | opportunity | elimination | employers | countries | contractor | party | context | principles | vulnerable | services | positions | categories | recommendations | breaches | ability |
| turnover | | consultation | control | accordance | bodies | expected | united | ILO | persons | society | cultural | contracts | actual | business | OECD | cycle | responsible | complains | foundation |
|  | | significant | ill | performance | gender | women |  |  |  |  | identified |  |  | relationship | designed | development |  | losses | national |
|  | | notice | hazards | career | indicators |  |  |  |  |  |  |  |  |  |  |  |  | substantiated |  |
|  | |  | workplace | assistance |  |  |  |  |  |  |  |  |  |  |  |  |  |  |  |

Remarks: International Labour Organization is abbreviated as ILO while Organization for Economic Co-operation and Development is abbreviated as “OECD”.
