# Supplementary material for: Changing trends of corporate social responsibility reporting in the world-leading airlines
Source: PLoS One. 2020 Jun 8;15(6):e0234258. doi: 10.1371/journal.pone.0234258 (PMC7279591; doi:10.1371/journal.pone.0234258)
Supplement: S5 Appendix — (DOCX) [file pone.0234258.s008.docx]

**Appendix 5. The categorization of EU based airlines and APEC based airlines**

| **EU based airlines** | **APEC based airlines** |
| --- | --- |
| Air France-KLM | Aeroflot |
| Alitalia | Air Canada |
| Croatia | Air China |
| Finnair | American Airlines |
| Lufthansa | ANA |
| SAS | Cathay Pacific |
|  | China Airlines |
|  | Delta Air Lines |
|  | Garuda Indonesia |
|  | Japan Airlines |
|  | Korean Airlines |
|  | Latam Airlines |
|  | Singapore Airlines |
